# Fresh aquaculture sludge management with black soldier fly (*Hermetia illucens* L.) larvae: investigation on bioconversion performances

**DOI:** 10.1038/s41598-023-48061-0

**Published:** 2023-11-28

**Authors:** Giacomo Rossi, Shikha Ojha, Andreas Müller-Belecke, Oliver K. Schlüter

**Affiliations:** 1 Department of Systems Process Engineering, Leibniz Institute for Agricultural Engineering and Bioeconomy (ATB), Max-Eyth-Allee 100, 14469 Potsdam, Germany; 2 Department of Land Sciences, School of Science and Computing, South East Technological University, Cork Road, Waterford, X91 K0EK Ireland; 3 Institute of Inland Fisheries Potsdam-Sacrow, Im Königswald 2, 14469 Potsdam, Germany; 4 Department of Agricultural and Food Sciences, University of Bologna, Piazza Goidanich 60, 47521 Cesena, Italy

**Keywords:** Biomaterials - proteins, Environmental impact

## Abstract

Aquaculture solid waste (ASW) is a nutrient rich material that can pose a significant environment challenge if not properly managed. This study investigated the potential of black soldier fly (BSF) larvae in converting this waste into biomass. Five substrates comprising chicken feed supplemented with varying proportions of fresh ASW (0%, 25%, 50%, 75%, 100%) were formulated and evaluated for larval growth and waste bioconversion efficiency. High nutrients retention (N: 23.25 ± 1.40%; C: 21.94 ± 0.99%; S: 12.20 ± 1.33%) and feed conversion ratio (1.78 ± 0.08) were detected on substrate 100ASW, although the limited feeding rate (114.54 ± 5.38 mg dry substrate/larvae) and the high amount of indigestible fibres (ADF = 15.87 ± 0.24%; ADL = 6.36 ± 0.17%) were translated to low larval growth (final larval average weight: 66.17 ± 1.81 mg). Decreasing ASW content resulted in reduced fibres and ash, increase in non-fibrous carbohydrates and C/N ratio, and improved larval growth and substrate utilization. However, high larval metabolic activity suggested higher nutrients loss to the environment. Substrate 75ASW demonstrated the best performances in terms of larval production (final larval average weight: 176.30 ± 12.12 mg), waste reduction (substrate reduction corrected by percentage of ASW: 26.76 ± 0.86%) and nutrients assimilation (N: 22.14 ± 1.14%; C: 15.29 ± 0.82%; S: 15.40 ± 0.99%). This substrate closely aligned with optimal BSF rearing substrates reported in literature. Overall, this study highlights the potential of BSF larvae in managing fresh ASW, offering a dual benefit of waste reduction and insect biomass production.

## Introduction

Aquaculture solid waste (ASW) management is one of the biggest environmental problems connected with the aquaculture sector^1^. Such waste (a.k.a. aquaculture sludge) is mainly composed of fish carcasses, uneaten feed, faecal drops and metabolic products^2^, and is rich in nitrogen, phosphorous and organic matter^3^. It can potentially be a serious threat for both, fish rearing performances and environmental sustainability. If not readily removed, such material may accumulate in the rearing pond causing eutrophication and generating greenhouse gas emission^4,5^.

Traditional intensive and semi-intensive aquaculture systems have low efficiency in terms of nutrient assimilation and recovery, resulting in high concentration of organic matter, nitrogen and phosphorous in the rearing water^1^. Innovative technologies, such as integrated multi-trophic aquaculture, aquaponic, biofloc technology and recirculating aquaculture system (RAS), which have the aim of solving these problems have been implemented^1^. The principle behind such systems is to use the aquaculture rearing waste and/or recycle the effluent water, which is cleaned, disinfected, re-oxygenated and reintroduced in the rearing pond. Although these technologies can significantly reduce the amount of effluent water discarded from the system, they cannot solve the production of aquaculture solid waste^2^.

One solution for combining waste treatment along with nutrient recovery may be the bioconversion of aquaculture sludge by insects. Black soldier fly (BSF, *Hermetia illucens*) larvae have been reported for being able to efficiently grow on organic wastes, such as manure and food waste, converting them in high quality proteins to be used as animal feed^6,7^. Additionally, the residual material after insect rearing (frass) may still be conveniently used as organic fertiliser in agriculture^8^, thus promoting a transition to the circular economy.

Despite the growing interest in using insects for waste recycling, only few studies have investigated the ability of BSF larvae in bio-converting ASW, so far. In a study from Schmitt et al.^9^, oven-dried aquaculture sludge from salmon farming was regenerated at 70% of moisture and tested for BSF larvae rearing. Although the authors concluded that ASW was suitable for BSF growth, insects’ performances were particularly low, suggesting the need to add other ingredients to the rearing substrate. A recent study from Liland et al.^10^ has shown that addition of chicken feed to the oven dried salmon sludge could ensure acceptable larval growth with a final larval weight stable at levels of sludge inclusion as high as 40%. However, the drying process applied to the sludge may have had a strong impact on the substrate quality, hiding the real potential of ASW for rearing BSF larvae. Moreover, the oven drying is an energy costly pre-process, which may limit the technical applicability of the material.

In the present study, the ability of BSF larvae to reduce and convert fresh ASW from pikeperch (*Sander lucioperca*) production was investigated. Intermediate aquaculture sludge discarded from the drum filter (i.e. before the bacterial treatment for NH_3_ removal) of a pilot scale RAS was mixed with commercial chicken feed (a common rearing substrate for BSF larvae^11^) in different ratios and larval growth and bioconversion performances were evaluated. This research aims to evaluate BSF larval growth and bioconversion under varying ASW/chicken feed mixtures ratios, contributing valuable insights to the sustainable ASW management.

## Results

### Chemical composition of substrates

All the tested substrate, except 100ASW, showed a similar DM with values ranged between 65 and 70%. Substrate 100ASW contained significantly lower DM, with a moisture level of 92.05 ± 0.10% (Table 1).

**Table 1:** Composition and final dry matter (% of fresh matter) of the experimental substrates.

Treatment	ASW (%)	CF (%)	ASW (g)	CF (g)	Water (g)	Total wet weight (g)	Dry matter (% fresh matter)*
0ASW	0	100	0	99.47	200.53	300.00	36.53 ± 0.13
25ASW	25	75	41.76	125.28	132.96	300.00	30.23 ± 0.12
50ASW	50	50	117.31	117.31	65.38	300.00	29.10 ± 0.04
75ASW	75	25	225	75.00	0.00	300.00	29.94 ± 0.66
100ASW	100	0	300	0.00	0.00	300.00	7.95 ± 0.06

*Dry matter is expressed as mean ± standard error of 3 repetitions.

*ASW* aquaculture solid waste, *CF* chicken feed.

**Figure 1:**
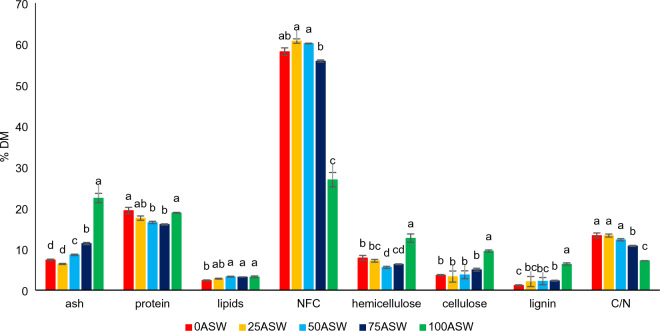
Chemical composition (% DM) of diets used for BSF larvae rearing. Error bars correspond to the standard error computed on 3 replicates/treatment. Different letters on top of the bars within the same parameters indicate significant difference (P < 0.05).

In terms of composition (Fig. 1), CF acted as the main source of carbohydrates, with the 0ASW diet exhibiting an NFC level of 58.17 ± 0.91%, which was similar to substrates 25ASW and 50ASW, but significantly higher than treatment 75ASW and 100ASW (P = 0.006). Inclusion of ASW in diet led to a progressive increase in ash (Beta regression, P = 0.006), hemicellulose (P < 0.001), cellulose (P < 0.001), and lignin (P = 0.006). Lipid contents were found similar in 25ASW, 50ASW, 75ASW, 100ASW and slightly lower value was observed in 0ASW (P = 0.003). Carbon/Nitrogen ratio gradually decreased from approximately 13 (0ASW, 25ASW, 50ASW) to 10.78 (75ASW), reaching to 7.17 in the substrate 100ASW (P = 0.006). The estimated level in proteins was similar in 0ASW (19.40 ± 0.79%DM) and 100ASW (18.85 ± 0.10%DM), although some differences were detected between these two substrates and treatments 25ASW (17.56 ± 0.52%DM), 50ASW (16.51 ± 0.28%DM) and 75ASW (16.01 ± 0.14%DM) (P = 0.001).

### Larval growth and bioconversion performances

Larval performances in terms of ability to grow, convert the diet in larval body mass and reduce the overall substrate, are presented in Table 2. Inclusion of ASW in diet did not showed any adverse effect on larval survival, with value ranged between 75.83 (25ASW) and 89.00% (50ASW). Slower development was observed for larvae housed on substrates 75ASW and 100ASW (P = 0.002), where the first pre-pupae was observed 2 days later than in the other substrates.

**Table 2:** Growth and bioconversion indexes of BSF larvae reared on the experimental diets (mean ± standard error; n = 3).

Parameter	0ASW	25ASW	50ASW	75ASW	100ASW
Survival (%)	83.00 ± 4.80	75.83 ± 1.83	89.00 ± 1.53	84.50 ± 5.00	88.74 ± 1.13
Development (days)	8.00 ± 0.00	9.33 ± 0.67	8.67 ± 0.67	10.00 ± 0.00*	10.00 ± 0.00*
GW (mg)	0.07 ± 0.01 b	0.06 ± 0.00 b	0.08 ± 0.01 b	0.17 ± 0.01 a	0.06 ± 0.00 b
AF (g)	47.64 ± 2.42 a	25.49 ± 2.68 b	21.22 ± 2.44 b	32.14 ± 1.75 b	5.28 ± 0.07 c
FCR	14.08 ± 2.69 a	9.18 ± 1.84 ab	4.55 ± 0.89 bc	2.80 ± 0.08 bc	1.78 ± 0.08 c
BCR (%)	4.04 ± 0.72 b	3.23 ± 0.30 b	4.89 ± 0.44 b	9.68 ± 0.52 a	3.92 ± 0.20 b
Reduction (%)	43.43 ± 2.10 a	28.07 ± 2.92 bc	23.88 ± 2.79 bc	35.68 ± 1.14 ab	22.11 ± 0.16 c

*ASW* aquaculture solid waste, *GW* gained weight, *AF* assimilated feed, *FCR* feed conversion ratio, *BCR* biomass conversion rate.

Values within a row followed by different letters are significant different (P < 0.05).

Survival and development values followed by a star (*) are significant different from the control (0ASW) (GLM + Dunnett’s test).

All the measured parameters are referred to the final larvae.

**Figure 2:**
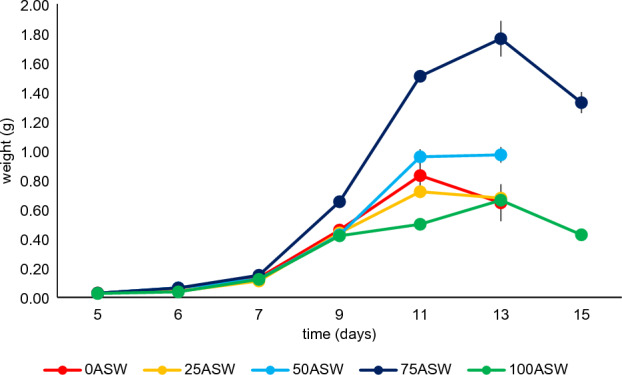
Averaged weight increase trend of 10 BSF larvae reared on the experimental substrates. Error bars correspond to the standard error computed on 3 replicates/treatment.

Average gained weight (GW) ranged between 0.06 and 0.08 g, except for treatment 75ASW where an increase of 0.17 g was observed (P < 0.001). Trend of weight increase overtime was similar in all the treatments (Fig. 2), with an exponential tendency observed during the first 11 (0ASW, 25ASW, 50ASW) or 13 days (75ASW), followed by a stationary (50ASW) or decreasing phase. Two steps growth was observed in treatment 100ASW, where larvae raised faster during the first 9 days and slower between day 9 and day 11.

As shown in Table 2, treatment 75ASW resulted in significantly higher BCR than the other treatments (P < 0.001), with double value than the control (0ASW). A clear decreasing trend was observed for AF and FCR with an increasing percentage of ASW inclusion in the diet, which decreased from 47.64 g and 14.08 (0ASW) to 5.28 g and 1.78 (100ASW), respectively. No clear trend was observed in terms of substrate reduction, with treatments 0ASW (43.43%) and 75ASW (35.68%) showing significantly higher values than the other substrates (P < 0.001).

Larval activity on the substrate led to reduction in DM, VS, hemicellulose, cellulose, C, TOC, N, S and P (Table 3). The highest reductions were always observed in treatments 0ASW and 75ASW, regardless the considered nutrient. High reduction in K and NO_3_-N were also observed, while concentration of NH_4_-N increased in all the treatments, except in treatment 100ASW where a reduction of 75% (fresh matter bases) was noticed. pH increasing was observed in all the treatments. Initial substrates had a pH ranged between 4.89 and 6.63, with substrate 100ASW showing the highest pH. The lowest pH was detected in substrate 25ASW, followed by substrate 50ASW (4.92), 75ASW (5.23) and 0ASW (5.99). Regardless of the treatment, pH of the final material was close to neutral, being ranged between 6.62 (25ASW) and 7.33 (100ASW). pH 7 (7.03) was observed in frass 0ASW, while similar pH was recorded from frass 50ASW (7.22) and 75ASW (7.33).

**Table 3:** Reduction in chemical fractions (% of fresh matter) and pH changes after the BSF-mediated substrate bioconversion.

Parameter	0ASW	25ASW	50ASW	75ASW	100ASW
VS	43.16 ± 2.26 a	30.46 ± 2.17 b	27.68 ± 2.65 b	38.89 ± 0.87 ab	30.65 ± 1.34 ab
N	45.16 ± 5.43 ab	19.82 ± 2.67 b	33.38 ± 9.16 ab	54.68 ± 0.50 a	40.88 ± 0.44 ab
C	43.74 ± 1.93 a	31.41 ± 2.55 b	29.12 ± 2.53 b	40.91 ± 0.75 ab	34.34 ± 0.39 ab
S	40.37 ± 1.61 a	13.64 ± 2.13 c	20.18 ± 6.96 bc	30.19 ± 0.56 ab	22.67 ± 0.97 abc
TOC	42.10 ± 2.36 a	28.14 ± 4.09 ab	26.26 ± 3.59 b	42.74 ± 2.06 a	34.72 ± 1.05 ab
NH_4_-N	− 204.66 ± 31.32 c	− 96.70 ± 28.63 b	− 93.15 ± 4.46 b	-33.46 ± 1.38 b	75.28 ± 0.90 a
NO_3_-N	82.29 ± 3.55 ab	60.92 ± 2.09 b	69.47 ± 3.60 b	87.83 ± 2.45 a	95.82 ± 0.57 a
P	28.58 ± 6.43 a	6.50 ± 1.69 b	4.82 ± 4.91 b	6.48 ± 2.45 b	5.57 ± 1.40 b
K	84.52 ± 2.14 b	89.17 ± 0.42 b	87.22 ± 0.14 b	89.38 ± 1.04 b	99.70 ± 0.01 a
Hemicellulose	37.16 ± 4.22 a	22.02 ± 2.46 ab	15.56 ± 3.65 b	23.43 ± 1.16 ab	3.71 ± 0.57 c
Cellulose	27.45 ± 7.43 a	15.65 ± 2.29 b	10.36 ± 0.12 b	17.75 ± 1.86 b	9.93 ± 1.02 b
Lignin	27.93 ± 2.54 a	− 18.79 ± 8.85 c	11.35 ± 4.55 b	22.01 ± 3.89 a	− 0.79 ± 0.38 c
pH*	1.04 ± 0.19 b	1.73 ± 0.13 a	2.30 ± 0.22 a	2.00 ± 0.06 a	0.70 ± 0.02 b

*ASW* aquaculture solid waste, *VS* volatile solids, *N* total nitrogen, *C* total carbon, *S* total sulphur, *TOC* total organic carbon, *NH*_*4*_*-N* ammonium nitrogen, *NO*_*3*_*-N* nitric nitrogen, *P* elemental phosphorous, *K* elemental potassium.

Values within a row followed by different letters are significant different (P < 0.05).

*pH changes computed as difference between pH of frass and pH of substrates.

**Figure 3:**
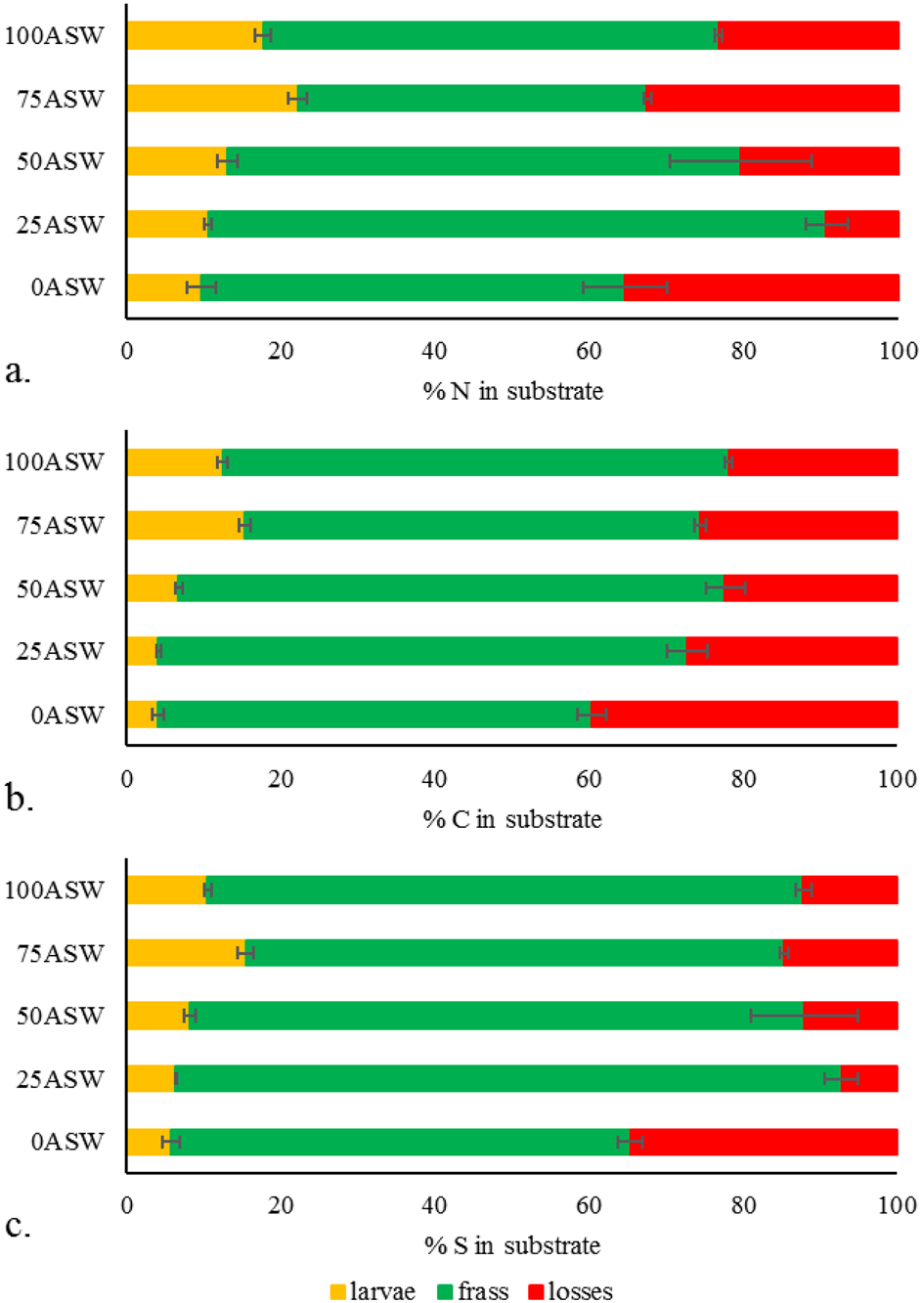
Distribution (%) of nitrogen (**a**), carbon (**b**) and sulphur (**c**) between larvae and frass as results from the mass balance approach. Error bars correspond to the standard error computed on 3 replicates/treatment. Losses represent the percentage of each element (nitrogen, carbon or sulphur) not recovered in larvae and frass (i.e. emitted as gas in the environment).

The mass balance of nitrogen, carbon and sulphur and relative nutrient retention from insects are shown in Fig. 3. Irrespective by the nutrient, the highest retention was always detected in treatment 75ASW followed by treatment 100ASW, whose values were significantly higher than the other treatments (ANOVA, P < 0.05). No statistically significant differences were observed between 0ASW, 25ASW and 50ASW. The amounts of lost nutrients were conspicuously higher in treatment 0ASW than in the others, except for nitrogen where no differences were detected between 0ASW (35.44%) and 75ASW (32.53%). Lower nitrogen loses were observed in treatments 50ASW (20.37%) and 100ASW (23.25%), while substrate 25ASW presented very limited loses (9.25%).

### Nutritional composition of insects

Chemical composition of BSF larvae growth on the tested substrates is shown in Table 4. DM was always close to 30%, with the only exception of larvae 75ASW and 100ASW, which showed significantly higher and lower DM (P < 0.001). VS appeared for being stable among the different treatments containing CF, with significantly lower values only detected in treatment 100ASW (P < 0.001). Protein, lipids and NFC varied according with the treatment, with the highest values detected in larvae 25ASW for protein, 50ASW and 75ASW for lipids, 75ASW and 100ASW for NFC, while significantly lower values were measured in larvae 75ASW for protein (P = 0.006) and 100ASW for lipids (P < 0.001). Fibres content was stable between treatments, except for larvae 100ASW, which presented a significantly lower amount of NDF (P = 0.012) and higher amount of ADL (P = 0.038). No statistically relevant differences were observed in ADF concentration.

**Table 4:** Chemical composition of BSF larvae reared on the different experimental substrates (mean ± Standard Error, n = 3).

Parameter	0ASW	25ASW	50ASW	75ASW	100ASW
DM	29.90 ± 0.45 b	29.88 ± 1.48 b	32.82 ± 0.93 b	39.53 ± 0.35 a	25.32 ± 0.57 c
VS	87.60 ± 0.52 a	89.71 ± 2.15 a	88.20 ± 0.46 a	86.27 ± 0.36 a	80.62 ± 0.40 b
Protein	43.13 ± 0.82 b	48.52 ± 3.20 a	40.25 ± 1.02 b	33.90 ± 0.46 c	40.60 ± 0.60 b
Lipids	13.40 ± 0.52 ab	10.09 ± 1.53 b	16.80 ± 0.68 a	15.63 ± 0.91 a	2.11 ± 0.15 c
NFC	13.13 ± 1.65 b	11.65 ± 1.53 b	11.30 ± 0.85 b	20.34 ± 0.74 a	22.94 ± 0.56 a
NDF	17.94 ± 0.48 ab	19.45 ± 1.42 a	19.85 ± 1.08 a	16.40 ± 0.36 ab	14.97 ± 0.36 b
ADF	8.24 ± 0.09 a	8.35 ± 1.21 a	9.51 ± 0.36 a	8.12 ± 0.18 a	8.31 ± 0.29 a
ADL	1.75 ± 0.15 ab	1.25 ± 0.12 b	1.83 ± 0.41 ab	1.33 ± 0.25 ab	2.45 ± 0.18 a

The values are in % of dry matter.

*ASW* aquaculture solid waste, *DM* dry matter, *VS* volatile solids, *NFC* not fibrous carbohydrates, *NDF* neutral detergent fibres, *ADF* acid detergent fibres, *ADL* acid detergent lignin, *C/N* carbon to nitrogen ratio.

Values within a row followed by different letters are significant different (P < 0.05).

### Nutritional composition of frass

Table 5 displays the chemical composition of frass. Although the substrates had different DM, no significant differences were detected on DM between frass from the different treatments (P = 0.122). Compared with the others, frass from treatment 100ASW appeared for being significant poorer in VS (72.70%), C (38.60%), TOC (34.23%) and K (0.90 g/kg FM). On the other hand, the same frass were richer in fibres, N (4.85%) and P (25.62 g/kg FM). No significant differences were detected on NO_3_-N, while significant higher amount of NH_4_-N was recorded in treatment 75ASW (5003.94 mg/kg FM) than in the other treatments (P < 0.001). The lowest amount of NH_4_-N was measured in frass 0ASW (1508.18 mg/kg FM), which presented also a high C/N ratio (13.82). No significant differences were detected between frass 0ASW and 75ASW in terms of N and C/N ratio, while significant lower amounts of C and TOC, and higher amounts of S and P were measured in frass 75ASW than in 0ASW.

**Table 5:** Chemical composition and pH of frass generated by BSF larvae reared on the different substrates (mean ± standard error, n = 3).

Parameter	0ASW	25ASW	50ASW	75ASW	100ASW
DM (% FM)	85.25 ± 1.01 a	85.79 ± 0.39 a	82.47 ± 1.77 a	83.55 ± 1.61 a	87.03 ± 0.14 a
VS	93.22 ± 0.45 a	91.57 ± 0.85 ab	89.76 ± 0.04 b	86.28 ± 0.27 c	72.70 ± 0.24 d
Lipids	0.73 ± 0.10 b	0.96 ± 0.14 b	1.95 ± 0.08 a	1.98 ± 0.20 a	0.70 ± 0.03 b
Hemicellulose	8.72 ± 0.38 b	7.84 ± 0.07 c	6.34 ± 0.07 d	7.68 ± 0.08 c	15.88 ± 0.10 a
Cellulose	4.72 ± 0.56 c	4.88 ± 0.12 c	5.22 ± 0.17 c	6.94 ± 0.08 b	11.58 ± 0.05 a
Lignin	1.51 ± 0.18 c	2.12 ± 0.16 bc	2.05 ± 0.09 bc	2.85 ± 0.18 b	8.23 ± 0.36 a
Nitrogen	3.29 ± 0.21 c	3.91 ± 0.02 b	3.36 ± 0.31 c	3.08 ± 0.10 c	4.85 ± 0.02 a
Carbon	45.06 ± 0.02 a	44.49 ± 0.17 a	44.28 ± 0.17 ab	43.27 ± 0.82 b	38.60 ± 0.06 c
TOC	40.24 ± 0.21 a	39.52 ± 0.73 a	40.00 ± 0.40 a	37.30 ± 0.85 c	34.23 ± 0.09 d
Sulphur	0.32 ± 0.02 c	0.36 ± 0.00 c	0.37 ± 0.01 c	0.46 ± 0.01 b	0.86 ± 0.00 a
C/N	13.82 ± 0.92 a	11.37 ± 0.11 b	13.43 ± 1.27 ab	14.06 ± 0.17 a	7.96 ± 0.03 c
NH_4_ (mg/kg FM)	1508.18 ± 149.64 c	2035.26 ± 229.27 c	3287.58 ± 199.03 b	5003.94 ± 48.71 a	3243.12 ± 104.28 b
NO_3_ (mg/kg FM)	1.13 ± 0.30 a	2.00 ± 0.16 a	3.05 ± 1.76 a	0.68 ± 0.09 a	0.85 ± 0.02 a
P (g/kg FM)	5.33 ± 0.55 d	6.65 ± 0.23cd	7.37 ± 0.30 c	11.51 ± 0.24 b	25.62 ± 0.17 a
K (g/kg FM)	13.00 ± 1.72 a	12.81 ± 0.65 a	12.66 ± 0.18 a	11.18 ± 0.47 a	0.90 ± 0.03 b
pH	7.03 ± 0.19 ab	6.62 ± 0.13 b	7.22 ± 0.22 ab	7.23 ± 0.06 ab	7.33 ± 0.02 a

The values are in % of dry matter, unless otherwise specified.

*ASW* aquaculture solid waste, *DM* dry matter, *FM* fresh matter, *VS* volatile solids, *TOC* total organic carbon, *C* total carbon, *N* total nitrogen, *NH*_*4*_ ammonium nitrogen, *NO*_*3*_ nitric nitrogen, *P* elemental phosphorous, *K* elemental potassium.

Values within a row followed by different letters are significant different (P < 0.05).

## Discussion

Based on the authors’ knowledge, this is the first study investigating the potential of BSF for reducing fresh ASW. Recently, Liland et al.^10^ and Schmitt et al.^9^ tested the use of ASW for rearing BSF, with focus on growth and chemical quality of insects, without considering the aspects related to the waste conversion process. Additionally, an oven drying process was applied to ASW in those studies. While drying might be needed to control substrate moisture more effectively^12^, it is generally unfavourable in terms of economical and quality considerations, being responsible for vitamins degradation and microbial inactivation^13^. Considering that the present study utilized fresh ASW, such negative effects were not expected. Additionally, in the present study, ASW was collected daily, ensuring that it did not accumulated over time. According to Banks et al.^14^, accumulation of faeces may lead to anoxic conditions which might lead to reduced larval feeding and survival.

### Effect of different substrates on BSF performances

Results from this study showed that BSF larvae can effectively convert fresh ASW, seize nutrients from it and therefore reduce its polluting potential. Although treatment with pure ASW (100ASW) yielded the lowest substrate reduction, AF and insect growth, a comprehensive analysis of nutrient reduction and retention reveals a noteworthy assimilation by the BSF larvae. The observed low performances in the substrate 100ASW could be attributed to the feeding rate based on dry matter content. In the present experiment, a similar amount of fresh feed (300 g) was provided in each treatment. It resulted in a constant feed volume (substrate height ~ 2 cm) and larval density (0.6 larvae/cm^2^), two parameters considered crucial for the BSF larvae rearing^15^. As pure ASW presented a very limited DM (7.95 ± 0.06%), the amount of energy and nutrients provided to the larvae in this treatment was low and might not have been sufficient for supporting larval growth. Diener et al.^16^ and Manurung et al.^17^ have also reported lower larval weight and longer development time when limited feed was offered. However, despite the extremely limited amount of dry substrate, high larval survival was recorded, suggesting that the amount of provided diet was still enough for reaching the critical weight and supporting larval development^18^. It might be due to the high amount of nitrogen and protein content of the ASW. It is well known that nitrogen is crucial for building the body structure and reaching the larval critical weight^19–21^. As protein concentration of larvae growth on treatment 100ASW was similar to the larvae 0ASW, it can be stated that sufficient amount of protein was provided to the larvae in this treatment. When the nitrogen requirement is fulfilled, BSF larvae start accumulating lipids as energy reserve (fattening), resulting in higher final weight^22,23^. The low lipid concentration in larvae 100ASW suggests that very limited amount of energy was accumulated in these larvae. As a balanced C/N ratio and nutrients level are responsible for reaching high larval weight^24,25^, increasing the energy level of the feed should be accompanied by a proportional increase of the nitrogen content. However, when the critical weight is reached, the consumption of nitrogen and carbon is not balanced, resulting in lower larval nutrient retention and therefore higher amount of nutrients volatilization^26,27^. Recent research on earthworms confirms such findings^28^, suggesting that low feeding rate in waste management should be pursued whether the aim is to reduce the overall environmental impact of the system.

The high nutrients retention and reduction observed in 100ASW were not accompanied by a similarly high dry substrate reduction. It suggested that despite the low feeding rate, the amount of substrate used by BSF larvae was very low. The main reason for it could be found on the high level of indigestible fibres (i.e. cellulose and lignin) detected in substrate 100ASW^29^. Similar results were obtained by Rehman et al.^30^ for BSF larvae raised on pure cow manure. Indeed, although such fibres can be degraded by microorganisms living in the gut of BSF larvae^31^, very limited hemicellulose and cellulose degradation and total absence of lignin degradation were noticed in treatment 100ASW. Addition of CF to the ASW led to an increasing concentration of carbohydrates and total VS, while percentage of fibres decreased. Accordingly, C/N ratio as well as carbohydrate to protein ratio increased reaching values of 10.78–13.33 and 3.01–3.49, respectively. While reduction of fibre content in substrate was beneficial for promoting direct larval feed consume^32–34^, more balanced nutrient composition could favourite a better microbial degradation of the material that cannot directly be digested by BSF larvae^24^. It followed that the substrate with lower fibre content and the most balanced C/N ratio, i.e. 0ASW, was the substrate better used by the BSF larvae, explaining the significantly higher substrate reduction and AF observed in this treatment. Nevertheless, the high FCR (i.e. amount of feed needed for increasing the larvae weight of 1 g) and low BCR (i.e. amount of feed converted in larval biomass) computed for treatment 0ASW indicated that BSF larvae did not efficiently consumed this substrate. Suitable explanation could be found in the scarce homogeneity of the substrate^35^. As CF used in our experiment presented high dry matter (90.16 ± 0.16%), a significant amount of water was added. It resulted in an observable water phase on top of the substrate that might have been hindered oxygen dissolution, resulting in an anaerobic environment responsible for low larval activities^12,36^. Increasing the amount of ASW in substrates resulted in a reduction of extra water added, as the ASW supplied most of the moisture in these substrates. As consequence, an improvement on the substrate structure could be assumed, explaining the better substrate to biomass conversion (lower FCR and higher BCR) detected in substrates 25ASW, 50ASW and 75ASW. However, unlike to our expectation, no clear trend was observed between ASW in diet and larvae performances. The only treatment that resulted significantly better was indeed the treatment 75ASW, which gave the heaviest larvae. Compared with 0ASW, 25ASW and 50ASW diets, the 75ASW substrate showed higher lipids and lower VS, protein and NFC. The contents of N and NH_4_-N were significantly higher, while the C/N ratio was lower. Altogether, such conditions resulted in a significant lower FCR and higher BCR which were comparable to pure poultry manure or mixtures of dairy and chicken manures^37^. It suggests that reduction in carbohydrate/protein ratio as well as C/N ratio of the substrate might be an interesting strategy for improving BSF larvae production^19^, although higher C and N losses and, therefore, higher environmental impact of the process should be considered^38,39^.

### Effect of different substrates on BSF larvae nutritional composition

Although the aim of this study was to investigate the ability of BSF larvae in growing on ASW based substrate and reducing the environmental impact of the aquaculture sector, an important aspect that should always be considered is the quality of the reared insects. Different studies have shown that quality of harvested insects is strictly dependent on the nutritional composition of the diet used as rearing substrate^37^. However, different nutrients show different patterns, with crude protein being more stable than lipids and ash^40^. In the present study, protein content of larvae varied from 33 to 50% (DM basis). The lowest protein content in 75ASW could be attributed to relative higher carbohydrates and lipids proportion, as well as the possible proximity to the pupation stage^41^. Specifically, the insects were harvested after observing the first pre-pupae. It means that a large number of larvae could not have yet reached the pre-pupal stage, partially hindering the results^42^. Lipids, which are accumulated as energy reservoir in the insect fat body, were constant, although significant different, between all the treatments, except for larvae 100ASW, where only 2% of lipids were detected. As already stated, feeding rate in this treatment was not enough for supporting BSF fattening, explaining the extremely low amount of fat accumulated. Interestingly, although larvae 75ASW were significantly heavier than the others, they did not show the highest lipid content. However, the same larvae displayed high NFC level. Since lipids can be synthesized from carbohydrates, it was possible that higher lipid concentration might be reached in a later stage. Ash content strictly followed the trend observed in the diets. It is consistent with previous findings^9,10,41^ and might be explained by the presence of residual feed in the larval gastro-intestinal tract^13^.

### Effect of different substrates on frass quality

Frass are the secondary material generated by insect-based waste management system. They consist of a nutrient rich material that, whether not correctly handled, may results in an important source of environmental impact^43,44^. Current practices include use of the frass as soil conditioner/fertilizer in agriculture. Several studies have focused on the nutritional composition of this material, evaluating its main effects on plant growth and soil health^8,45^. Frass obtained in this study showed high content in nitrogen and potassium, with NPK ratio ranged between 5:1:1 (75ASW) and 6.5:1:2.5 (0ASW). The only exception was represented by frass generated from treatment 100ASW, which showed a significantly higher content in phosphorous, with NPK ratio of 35:28.5:1. These results confirm previous findings reviewed by Basri et al.^45^, which showed that diets richer in protein generated frass richer in nitrogen, while diets richer in carbohydrates produces frass with low phosphorous concentration. As all the substrates used in the present study were rich in proteins, nitrogen-rich frass could be expected. On the other hand, as all the substrate except 100ASW were rich in carbohydrates, it is reasonable that the only frass with high amount of phosphorous was the one from treatment 100ASW. Additionally, it is interesting to notice that absolute amount of these nutrients followed a clear trend among treatments with substrates richer in ASW producing frass richer in nitrogen and phosphorous and poorer in potassium. However, no similar trends were observed in the initial substrates. As phosphorous and potassium are more stable than nitrogen in the substrates^29^, it can be speculated that assimilation of such nutrients by BSF larvae depends on the physiological status of the larvae, resulting in different concentrations in the obtained frass. Concerning the nitrogen content, a distinction between ammonium nitrogen and nitric nitrogen should always be done. Regardless of the treatment, frass obtained in our study appeared as an important source of ammonium nitrogen. Such nitrogen form is known for being more stable within the soil, being less available for plant absorption and atmospheric volatilization^46^. Although higher level of ammonium nitrogen over nitric nitrogen can be preferred in terms of environmental impact reduction, the same condition indicates a low maturity of the frass^45^, which should go through a further composting process before being applied in agriculture^47^. This condition has been frequently observed in BSF mediated bioconversion processes and it is usually associated to the short rearing period^48^. As in the present study, the longest residence time was only 10 days, it is reasonable to think that frass were far from being mature for agriculture application. Low compost maturity can also be assumed by considering the C/N ratio. Such ratio, although in line with the values reported in literature for BSF frass^45^, was particularly low and not significantly different from the C/N ratio of the initial substrates. As stable compost should present a C/N ratio between 20 and 40 for not being toxic for plants^49^, and significant increase of C/N ratio from substrate to frass was only observed in treatment 75ASW (from 10.78 to 14.06), it can be concluded that a further composting process is warranted before they can be used in agriculture.

## Conclusion and future research direction

The present work clearly shows that fresh ASW can be used for BSF larvae rearing, combining biomass production and nutrients recovery with waste management and pollution reduction. Although the limited feeding ratio applied on treatment 100ASW did not allow us to draft definitive conclusions, the high larval survival recorded on such treatment, combined with a larval protein content similar to the other treatments, led to conclude that pure fresh ASW is potentially suitable for rearing BSF larvae. Additionally, the feed limitation of the aforementioned treatment suggests a better resource utilization by BSF larvae, resulting in lower nutrients loss and FCR, and higher amount of nutrients retention. Addition of chicken feed to the ASW led to reduction of ash and fibres, while easy to digest carbohydrates and C/N ratio increased. These conditions resulted in better larval growth, bioconversion ratio, feed assimilation and substrate reduction. However, the efficiency on using the available resources decreased as lower larval nutrients retention and higher nutrients loss were observed. The treatment 75ASW was capable of most effectively combining ASW reduction with larval biomass production and low environmental impact. Frass generated from the different treatments showed high content in nitrogen and potassium, while phosphorous was relevant only in frass from treatment 100ASW. The C/N ratio of frass was similar to the C/N ratio of substrates, suggesting that a further composting period was needed before their application in agriculture.

Future research should aim to evaluate whether a higher feeding rate can improve the larval growth on pure fresh ASW, as well as to explore alternative materials other than CF for improving and optimizing the insect-based aquaculture waste management process. Furthermore, the microbial and chemical safety of insects produced on ASW, as well as the effect of BSF frass as a fertilizer and different post-composting processes for their stabilization should be considered in order to promote the full utilization of BSF in the circular economy. Finally, the ability of BSF larvae to reduce the amount of nutrients lost in the environment by decreasing their feeding rate without negatively affecting the overall performance of the system deserves more attention.

## Methods

### Insects

BSF eggs laid on corrugated cardboard within 36 h were purchased from Hermetia Baruth GmbH (Baruth/Mark, Germany) and placed in 2 Litres 21 × 15 × 11 cm polypropylene transparent boxes (Ikea Deutschland GmbH & Co. KG, art. 603.591.52, Munich, Germany) for hatching. The boxes were placed inside a climate chamber set at 30 ± 1 °C, 70 ± 5% RH and photoperiod 0:24 (L:D). Young larvae were carefully handled with a camel brush and transferred in similar polypropylene box filled with chicken feed (CF) (all-mash A Mehl mit Cocc., Deuka companion GmbH & Co. KG, Dusseldorf, Germany) moistened with distilled water at 50% of moisture and housed in a climate chamber at 28 ± 1 °C, 65 ± 5% RH, 0:24 (L:D). Eggs started hatching approximately 24 h after their purchase, but only the larvae hatched after further 12 h (from 36 to 48 h from the purchase) were used in the experiment.

### Aquaculture waste

Aquaculture discharged water from pikeperch (*Sander lucioperca*) was provided by the Institute of Inland Fisheries (Potsdam-Sacrow, Germany). Six-month old fishes (n = 940, average body weight: 103 g) were reared in a semi-industrial scale RAS equipped with 6 polyethylene rearing tanks (volume 1.3 m^3^ each), a moving bed biofilm reactor (biofilm surface 1000 m^2^), automatic feeders (Pflanzer Fütterungssysteme, Germany) and a drum filter (Hydrotech HDF 801 1G, Sweden). The water was kept at constant temperature of 23 °C and pH of 6.8 and it was surface-illuminated 16 h/day with dimmed light of 5 lx. Fishes were fed with commercial aquafeed (Biomar Efico Sigma 870F 3 mm, Denmark) every 4 h during the illumination period. Daily feeding rate was set at 1.5% of the fish biomass, correspondent to 1.4–1.7 kg of feed per day. For each kg of administered feed, 380 L of make-up water were added.

Approximately 50 L of drum filter wastewater containing c.a. 0.02% of solids (fish faeces and feed residuals) were daily collected for 2 weeks (from Monday to Friday, total ~ 500 L). In order to reduce the water level, daily collected wastewater was submitted to concentration, consisting in sedimentation (1 h, room temperature), followed by filtration (Ø 0.2 mm) and centrifugation (10 min, 3900 rpm, 20 °C). The separated water was discarded and the resulting sludge (ASW, moisture 90.93 ± 0.26%) was kept frozen at − 18 °C until the beginning of the rearing experiment.

### Experimental set-up

Frozen ASW was thawed at room temperature and thoroughly hand-mixed. Based on a preliminary experiment, five experimental substrates consisting of CF (all-mash A Mehl mit Cocc., Deuka companion GmbH & Co. KG, Dusseldorf, Germany) supplemented with ASW in different percentages (0—control, 25, 50, 75 and 100%) were formulated by hand-mixing the two ingredients. Substrate moisture was adjusted at 70% (except 100% ASW) by adding distilled water when needed (Table 1). This moisture level was chosen based on the data published by Bekker et al.^12^, who observed better BSF larvae growth with moisture ranged between 65 and 75%.

For each treatment, three experimental boxes (three replicates/substrate) were filled with 300 g (wet weight) of substrate (bulk volume ~ 450 cm^3^) and exactly 200 5-day old larvae (larval density 0.6 larvae/cm^2^). The amount of diet and the larval density were chosen for avoiding anaerobic condition which may impact larval development and substrate reduction^15^. It resulted in a significantly lower amount of dry material provided in substrate 100ASW.

Experimental boxes consisted of 2 L 21 × 15 × 11 cm polypropylene transparent box covered with a polypropylene transparent lid (Ikea Deutschland GmbH & Co. KG, art. 503.617.92, Munich, Germany) equipped with a rectangular hole (15 × 10.5 cm) covered with polyester black mosquito net (1 × 1 mm mesh, HaGa-Welt GmbH & Co. KG, Nordstemmen, Germany). These boxes were placed in a climate chamber set at 27 ± 1 °C, 70 ± 5% RH, 0:24 (L:D). For each single box, rearing was stopped and larvae were harvested when the first pre-pupae was observed in that box. It resulted in different harvesting time for each box (within the same treatment). At harvesting, all pre-pupae and larvae from the selected box were handpicked with forceps, washed with distilled water, dried with paper towel and inactivated by freezing at − 20 °C for 1 h. Larvae/pre-pupae, frass and samples of initial substrates were kept frozen at − 30 °C until the chemical analysis.

### Growth and waste bioconversion performances

In order to evaluate the ability of BSF larvae to grow on pure or CF-supplemented ASW, 10 larvae were randomly sampled every second experimental day (day 0, 2, 4, 6, 8, 10), cleaned with towel paper and weighted (Sartorius CPA224S-0CE Analytical Balance, Sartorius AG, Goettingen, Germany).

Total larval biomass, larvae numbers as well as diet and frass weights were recorded at the beginning and at the end of the rearing period. In order to better understand the changes occurring on the material during the bioconversion process, pH of diets and frass were measured^30^. Briefly, 1 g of material was mixed with 10 g of distilled water (1:10 w/w), the mixtures was vortexed for 5 s, incubated for 1 h at room temperature and the pH was measured with a benchtop pH meter (SI Analytics Lab 850 pH-Meter, SI Analytics GmbH, Mainz, Germany).

Bioconversion and growth performances were evaluated by computing the following indexes^37,42^:

1. Larval survival (%) = (final larval number/initial larval number) × 100.
2. Development time (day) = larvae harvesting day − larvae seeding day.
3. Gained weight per larvae (GW; mg) = final larval fresh weight − initial larval fresh weight.
4. Assimilated feed (AF; g) = total substrate dry weight − total frass dry weight.
5. Feed conversion ratio (FCR) = (dry weight of ingested feed)/(final larvae dry weight − initial larvae dry weight).
6. Biomass conversion rate (BCR; %) = [(total final larvae fresh weight − total initial larvae fresh weight)/total substrate fresh weight] × 100.
7. Substrate reduction (%) = [(total substrate dry weight − total frass dry weight)/total substrate dry weight] x 100.

### Chemical analysis

Initial substrate, residual frass and pre-pupae were analysed for dry matter (DM), ash, total nitrogen (N), total carbon (C), total sulphur (S), crude lipids, neutral detergent fibre (NDF), acid detergent fibre (ADF) and acid detergent lignin (ADL). Furthermore, total organic carbon (TOC), elemental phosphorous (P), elemental potassium (K), contents of ammonium (NH_4_-N), nitrite (NO_2_-N) and nitrate (NO_3_-N) were quantified in substrates and frass.

DM was determined by oven drying at 105 °C overnight (NFTA 2.1.4 official moisture method). Lipids were quantified with the Soxhlet method, by using petroleum ether as extraction solvent (AOAC, 2000—method 960.30). N was quantified with the Kjeldahl method (AOAC, 2000—method 992.15) and crude protein (CP) were computed by applying the factors 5.70 for CF^50^, 3.11 for ASW^51^ and 4.76 for BSF larvae^52^. NDF and ADF were quantified by gravimetric methods through digestion of the milled sample at 100 °C with neutral detergent or acid detergent solution respectively, followed by oven drying for 3 h at 102 °C. ADL was determined after further digestion for 3 h in 70% H_2_SO_4_ and oven drying (2 h, 105 °C). Hemicellulose was computed as difference between NDF and ADF, while cellulose was calculated as ADF-ADL^53^. Ash content was quantified through incineration of the sample at 550 °C for 4 h (DIN 38414-EN 12879), and total volatile solids (VS) were calculated by difference between DM and ash.

C and S were analysed through a catalytic raw combustion of the homogenised sample, operated under oxygen supply by the elemental analyser VARIO EL (Elementar Analysensysteme GmbH, Langenselbold, Germany), while P and K were quantified through atomic absorption spectroscopy (iCE 3300 AAS, Thermo Fisher Scientific, Waltham, USA) performed on the microwave acid digested sample (VDLUFA method, volume 3-chapter 10). TOC was gravimetrically determined, evaluating the reduction of the acidic treated sample weighted after evaporation of CO_2_ at 1200 °C (DIN-EN 13137:2001-12). Finally, NH_4_-N, NO_2_-N and NO_3_-N were analysed with the ion chromatography system Dionex ICS-1000 (Thermo Fisher Scientific, Waltham, USA), equipped with IonPac AS9-HC (4 × 250 mm) separation column (Thermo Fisher Scientific, Waltham, USA) and ASRS-Ultra detector (Thermo Fisher Scientific, Waltham, USA) for anions (NO_3_^−^, NO_2_^-^) or IonPac CS16 (5 × 250 mm) separation column and CSRS-Ultra detector (Thermo Fisher Scientific, Waltham, USA) for cations (NH_4_^+^).

Additionally, not fibrous carbohydrates (NFC) were determined for insects and substrates by applying the following formula^26^:

(8) NFC = 100 − (crude proteins + crude lipids + NDF + ash).

Chemical analyses were performed on oven-dried samples (60 °C, 48 h). Substrates were analysed in triplicate, while chemical analyses of larvae and frass were conducted in singlet.

Nutrients retention of larvae was evaluated by computing the following index for N, C and S:

(9) Nutrient retention (%) = [(%Nutrient in larvae × DM larvae)/(%Nutrient in substrate × DM substrate)] × 100.

A mass balance approach was applied for evaluating the overall conversion efficiency of the process.

### Statistical analysis

Statistical analyses were conducted in R software version 4.1.0^54^. Chemical composition, nutrient reduction and nutrient retention indexes were subjected to one-way analysis of variance (ANOVA). Normality and homoscedasticity of models residuals were evaluated by Shapiro–Wilk test and Levene test (package “car”) respectively. Beta regression (link logit) was applied when absence of normality was detected.

One-way analysis of covariance (ANCOVA) with development time as covariate, was used for comparing growth and bioconversion performances. Significance level was always set at 0.05 and Tukey HSD test for multiple comparison (package “multcomp”) was applied when significant differences were detected.

Larvae survival was analysed through general linear model (package “emmeans”) with binomial distribution (link logit), while general linear model with gamma distribution (link inverse) was used for modelling the development time. In both cases, Dunnett’s contrasts test (package “multcomp”) at significance level of 0.05 was used for computing the differences among treatments.

Generalised linear mixed model (GLMM, package “lme4”) with gamma-log link and substrate replicates as random effect (observation nested within the same box) followed by pairwise Tukey test with a significance level of 0.05 was used for evaluating the larvae growth over time^55,56^.

### Ethics approval and consent to participate

This study was approved by the regional food and feed safety authorities “327 Bereich Veterinär- und Lebensmittelüberwachung der Stadtverwaltung der Landeshauptstadt Potsdam, Geschäftsbereich Ordnung, Sicherheit, Soziales und Gesundheit, Fachbereich Ordnung, Sicherheit und Gesundheit” Brandenburg, Germany. The fish rearing was conducted by the Institut für Binnenfisherei e.V. Potsdam-Sacrow, authorisation number 386-1-384 issued by the Fachbereich Ordung, Siecherheit und Gesundheit, Bereich Veterinär-und Lebensmittelüberwachung—Municipality of Potsdam.

### Compliance with relevant guidelines and regulations

Animal housing, feeding experiment, sampling, killing operations, samples processing, animal waste management and analyses were carried out in accordance with the Deutsche Forschungsgemeinschaft (DFG) relevant guidelines and the Federal regulations (Article 20a of the Basic Law for the Federal Republic of Germany).

### Compliance with ARRIVE guidelines

The current study was carried out in compliance with the ARRIVE guidelines when relevant methods were applied.

## Data Availability

All data generated or analysed during this study are included in this published article.
